# Effect of total glycosides of *Cistanche deserticola* on the energy metabolism of human HepG2 cells

**DOI:** 10.3389/fnut.2023.1117364

**Published:** 2023-02-06

**Authors:** Duo Feng, Shi-qi Zhou, Ya-xi Zhou, Yong-jun Jiang, Qiao-di Sun, Wei Song, Qian-qian Cui, Wen-jie Yan, Jing Wang

**Affiliations:** ^1^College of Biochemical Engineering, Beijing Union University, Beijing, China; ^2^Beijing Key Laboratory of Bioactive Substances and Functional Food, College of Biochemical Engineering, Beijing Union University, Beijing, China; ^3^Institute of Food and Nutrition Development, Ministry of Agriculture and Rural Affairs, Beijing, China; ^4^Inner Mongolia Sankou Biotechnology Co., Ltd., Ordos City, Inner Mongolia, China

**Keywords:** *Cistanche deserticola*, total glycosides, HepG2 cells, mitochondrial respiration, glycolytic pressure

## Abstract

To study the anti-tumor effect of *Cistanche deserticola Y. Ma*, HepG2 cells were treated with 0, 3.5, 10.5, 21, 31.5, and 42 μg/ml of total glycosides (TG) from *Cistanche deserticola*. The HepG2 cell survival rate and 50% inhibition concentration (IC_50_) were detected using the CCK-8 method, and the level of reactive oxygen species (ROS) was detected by using a DCFH-DA fluorescence probe. Finally, a Seahorse XFe24 energy analyzer (Agilent, United States) was used to detect cell mitochondrial pressure and glycolytic pressure. The results showed that TG could reduce the survival rate of HepG2 cells and that the IC_50_ level was 35.28 μg/ml. With increasing TG concentration, the level of ROS showed a concentration-dependent upward trend. Energy metabolism showed that each dose group of TG could significantly decline the mitochondrial respiratory and glycolytic functions of HepG2 cells. In conclusion, TG could significantly inhibit the mitochondrial respiration and glycolysis functions of HepG2 cells, increase the level of ROS, and inhibit cell proliferation. Thus, this experiment pointed out that *Cistanche deserticola* can be used as a source of anti-cancer foods or drugs in the future. However, further studies on its mechanisms and clinical applications are needed.

## 1. Introduction

*Cistanche deserticola Y. Ma* is a homologous medicine and food substance. It is a perennial parasitic plant of the family Orobanchaceae and is called “desert ginseng” (1). Most importantly, nutrients such as fat and protein provided by *Cistanche* can meet the basic energy metabolism needs of normal human tissues and reduce the energy supply of tumor cells and thus achieve the purpose of inhibiting tumor cell growth. Moreover, *Cistanche* has the functions of antioxidation, suppression of inflammatory response, and enhancement of immunity (1, 2). Since the National Health Commission of the People's Republic of China and the State Administration for Market Regulation officially announced the production and operation of *Cistanche deserticola. Y. Ma* as a homologous substance of medicine and food was piloted in 2021, and there has been more in-depth research on its development.

Liver cancer is a common malignant tumor of the digestive system, and its mortality ranks at the forefront of malignant tumors (3, 4), which can be divided into primary and secondary. Nowadays, there is still a lack of safe and effective therapeutic drugs in the clinic. If sorafenib and other therapeutic drugs are taken for a long time, they will be prone to drug resistance and adverse reactions (5). Because the symptoms of early liver cancer are non-specific and most of the symptoms are in the middle and late stages, people are more active in the treatment of liver cancer with traditional Chinese medicine. Among patients with tumors and patients with unsatisfactory curative effects, such as chemoradiotherapy, traditional Chinese medicine reflects its advantages, such as, it can enhance immunity and be an adjuvant in early and medium-term tumor treatment.

In recent years, it has been reported that *Cistanche* has anticancer effects (6–11). Ye et al. (6) found that echinacoside extracted from *Cistanches herba* (*Cistanche salsa*) could inhibit the proliferation of HepG2 cells by reducing the expression of TREM2 and blocking the PI3K/Akt signal pathway. Li et al. (7) also found that echinacoside exerted an antitumor activity *via* the miR-503-3p/TGF-β1/Smad axis against liver cancer. Meanwhile, *Verbascoside* could regulate oxidative stress and apoptosis of HepG2 cells through STAT-3 and has the potential to be developed as a chemopreventive agent (8–10). Besides, researchers found that phenylethanol glycoside could also inhibit the proliferation effect of HepG2 cells (11). In addition, echinacoside inhibited the proliferation of renal cancer 786-O cells and SW480 colon cancer cells (12, 13). In another study, phenylethanol glycosides from *Cistanche* have also been shown to reduce liver injury in H22 tumor-bearing mice (14, 15) and to improve immune function by reducing AFP levels, thereby adversely affecting tumor growth (16). It is worth noting that altered energy metabolism is one of the characteristics of tumor cells, which is manifested as the fact that tumor cells use glucose glycolysis as a way of energy supply regardless of the aerobic or anaerobic environment, and this phenomenon is known as the “Warburg effect.” Therefore, it is theoretically possible to selectively starve tumor cells to limit tumor cell growth by limiting glucose uptake (17).

Although many studies have studied the anti-tumor effect of phenylethanol glycosides, such as *Echinacoside* and *Verbascoside*, the anti-tumor activity of TG (phenylethanoid glycosides, and other glycosides) (18) is not common. There are only a few studies on whether TG affects the energy metabolism of tumor cells. Thus, this experiment analyzed the relationship between energy metabolism, oxidative damage, and cell survival through glycolytic ability and mitochondrial pressure so as to explore the inhibitory activity of TG on HepG2 cells.

## 2. Materials and methods

### 2.1. Cell culture and treatment

HepG2 cells were purchased from the Cell Center of the Institute of Basic Medicine, Peking Union Medical University (Beijing, China), and DMEM complete medium containing 10% fetal bovine serum and 1% antibody (penicillin, streptomycin, and gentamicin) was used. The cells were cultured in a 37°C and 5% CO_2_ saturated humidity incubator (19). After the cells grew to a confluence of 80–90%, they were digested with 0.25% trypsin without EDTA and subcultured in 1:3. After 2–3 days of cell growth, the cells in the logarithmic growth stage were taken for a subculture or for subsequent experiments. In each experiment, HepG2 cells were exposed to different concentrations of TG.

Total glycosides of *Cistanche deserticola* were provided by Inner Mongolia Sankou Technology Co., Ltd (Ordos, Inner Mongolia, China) (20). TG was dissolved in a DMEM culture medium until the final concentrations were 0, 3.5, 10.5, 21, 31.5, and 42 μg/ml.

### 2.2. Effect of TG on HepG2 cell morphology

HepG2 cells in the logarithmic growth phase were inoculated into 24-well plates at a concentration of 5 × 10^4^/well. After the cells adhered to the wall, TG was added until the final concentrations were 0, 21, and 42 μg/ml and cultured for 24 h. Then, the cell morphology of each group was observed under the fluorescence microscope.

### 2.3. Effect of TG on the proliferation of HepG2 cells

The logarithm of the cell growth phase of HepG2 cells was taken as 1 × 10^4^/well, inoculated in 96-well plates, and each well was inoculated with 100 μl. After the cells adhered to the wall, they were dissolved in a serum-free DMEM medium such that the final drug concentrations were 0, 3.5, 10.5, 21, 31.5, and 42 μg/ml. Each concentration was allotted six multiple wells and then incubated at 37°C under 5% CO_2_ in an incubator for 24 h. Finally, to each well, 100 μl of DMEM culture medium containing 10% CCK8 solution was added, and then, the wells were incubated in an incubator for 30 min to detect the absorbance value (A) at 450 nm.

### 2.4. Measurement of intracellular ROS levels

The system of 2 ml per well containing 4 × 10^5^ cells was laid in a 6-well plate and placed in an incubator at 37°C under 5% CO_2_. After 24 h of treatment, the intracellular ROS level was measured. Then, 1 ml of DCFH-DA (10 μM) was added and incubated in the dark at 37°C for 30 min. Then, we washed them with PBS three times, and finally, the ROS activity was detected by flow cytometry.

### 2.5. Effect of TG on the energy metabolism of HepG2 cells

(A) Optimization of test conditions: HepG2 cells in the logarithmic growth period were selected and inoculated on the cell culture plate of a Seahorse XFe 24 bioenergy analyzer at 500 μl per well such that the number of cells was 1, 2, 4, and 8 ( × 10^4^). Five replicates of each group were made, of which only A1, B3, C4, and D6 were added with culture medium. (B) Mitochondrial pressure detection: a mitochondrial pressure kit was taken out in advance, which included oligomycin, carbonyl cyanide-trifluoromethoxy phenylhydrazone (FCCP), and rotenone/antimycin A (Rot/AA). They were diluted with the detection medium to make their concentrations 1 μM, 0.5 μM, and 0.5 μM, separately. Then, the hydrated probe plate was removed and added to ports A, B, and C, respectively, with a volume of 56 μl, 62 μl, and 69 μl. The oxygen consumption rate (OCR) values at different time intervals were measured to reflect the level of oxidative phosphorylation. (C) Glycolysis pressure detection: Glucose, oligomycin, and 2-deoxyglucose (2-DG) were diluted with the detection medium to make the concentrations 10 mM, 1 μM, and 50 mM, respectively. Then, the hydrated probe plate was removed and added to ports A, B, and C, respectively, with the aforementioned volumes. The extracellular acidification rate (ECAR) values at different time intervals were measured to reflect the glycolytic function of the cells (21).

### 2.6. Western blot

The treated cells were plated with 80 μl of lysis buffer (RIPA:PMSF = 100:1) that was used to lyse proteins, and then, sodium dodecyl sulfate-polyacrylamide gel electrophoresis (SDS-PAGE) was performed to separate the protein samples (50 μg loading volume per well). Later, the protein transfer to the PVDF membrane was accomplished using an ice bath, the proteins were blocked by BSA for 1 h at room temperature, and the primary antibodies (Bcl-2, Bax, caspase-3) were added proportionally and incubated overnight at 4°C. Afterward, the PVDF membranes were then incubated with diluted secondary antibodies for 1–2 h at room temperature. β-Actin was used as an internal reference protein, and PVDF membranes were developed and fixed to observe target protein expression changes (22, 23).

### 2.7. Statistical processing

The test data were expressed as $\bar{\chi }\pm SD$ (standard deviation) and analyzed by one-way analysis of variance (ANOVA) using SPSS 25.0 (SPSS, United States). Flowjo10 and Graphpad prism 8.0.2 were the software used to draw the figures. ^*^P < 0.05 and ^**^P < 0.01.

## 3. Results

### 3.1. Effect of TG on HepG2 cell morphology

**Figure 1:**
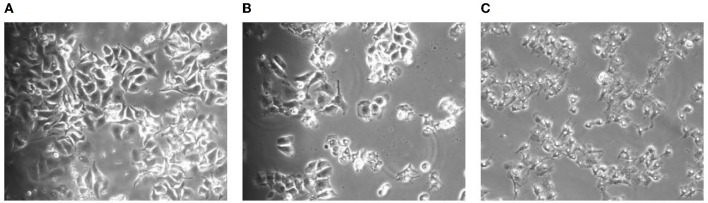
Morphological changes of the cells after TG treatment for 24 h. **(A)** Cell morphology after treatment with 0 μg/ml TG. **(B)** Cell morphology after treatment with 21 μg/ml TG. **(C)** Cell morphology after treatment with 42 μg/ml TG.

In the process of apoptosis, first, the cell volume will slowly decrease and deform and then cells growing closer to the wall will slowly shrink, become round, fall off, and result in chromosome pyknosis. Some nuclei will break, marginalize, and form apoptotic vesicles. It was observed from Figure 1 that, when HepG2 cells were treated with TG, the cell volume gradually decreased. It was intuitively observed that the nuclei of cells treated with 21 μg/ml of TG began to shrink and the volume became smaller. With 42 μg/ml TG, the cells were accompanied by floating and cell fragmentation; the cell membrane was completely broken, cracked, necrotic, with unclear boundary, and in a state of imminent collapse and death.

### 3.2. Effect of TG on the proliferation of HepG2 cells

To confirm the antitumor effect of TG *in vitro*, HepG2 cells were treated with a series of concentrations of TG (0, 3.5, 10.5, 21, 31.5, and 42 μg/ml) for 24 h. Then, the CCK8 assay method was used to explore the effect of TG on the proliferation of HepG2 cells.

**Figure 2:**
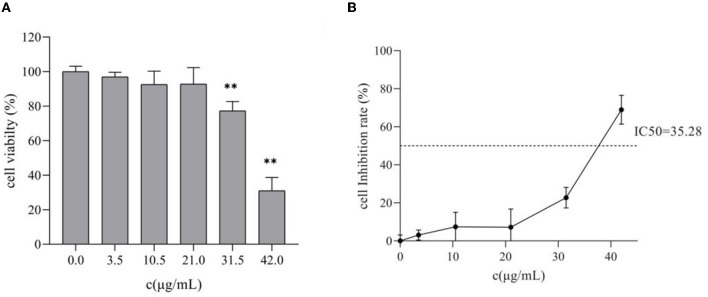
Effects of different concentrations of TG on the proliferation of HepG2 cells. **(A)** The survival rate of HepG2 cells. **(B)** The inhibition rate of HepG2 cells; IC_50_ represents the 50% inhibition of cell viability. **P < 0.01.

Figure 2 shows that the proliferation of HepG2 was affected to varying degrees after treatment with different concentrations of TG for 24 h. Compared with the cell survival rate of the control group, the cell survival rates of the treatment group were 96.95%, 92.59%, 92.78%, 77.28%, and 31.04%, respectively. With the increase in concentration, the cell survival rate showed a downward trend, but there was no significant difference (*P* > 0.05) in the concentrations of 3.5, 10.5, and 21 μg/ml, and the inhibition range was small. There were significant differences between the concentrations 31.5 and 42 μg/ml (*P* < 0.01), and the inhibition rate was as high as 68.96% at 42 μg/ml. Therefore, this experiment showed that TG could significantly inhibit the survival of HepG2 cells in a concentration-dependent manner, and further experiments may be needed to confirm the cytotoxicity of TG.

### 3.3. Measurement of intracellular ROS levels

**Figure 3:**
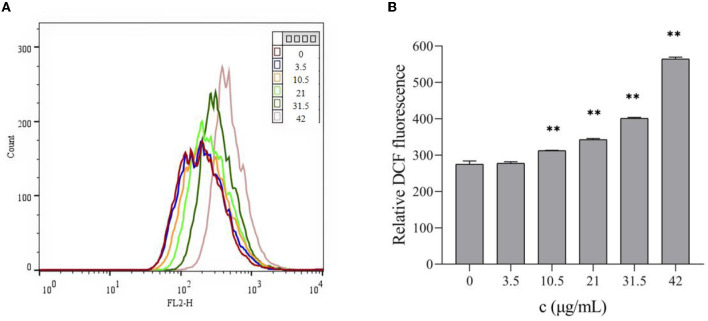
Effects of different concentrations of TG on the ROS levels of HepG2 cells. **(A)** The level of ROS was detected by flow cytometry. **(B)** Different concentrations of TG induced relative ROS production in HepG2 cells. **P < 0.01.

As shown in Figure 3, with the increase in TG concentration, higher relative contents of ROS were detected by the DCFH-DA fluorescence probe method, showing a dose gradient trend. After TG treatment for 24 h, the relative fluorescence intensity of DCF increased by 0.91%, 13.64%, 24.61%, 45.91%, and 105.33%, and there were highly significant differences at 10.5, 21, 31.5, and 42 μg/ml (*P* < 0.01). When TG concentration was 42 μg/ml, the maximum ROS accumulation level was observed to be about two times higher than that in the control group. Therefore, we speculated that the stable and high ROS production capacity may be the reason for the better cytotoxicity of TG. These results may also indicate a close relationship between ROS production ability and cell cytotoxicity.

### 3.4. Effect of TG on mitochondrial aerobic respiration of Hep2 cells

#### 3.4.1. Determination of cell number

**Figure 4:**
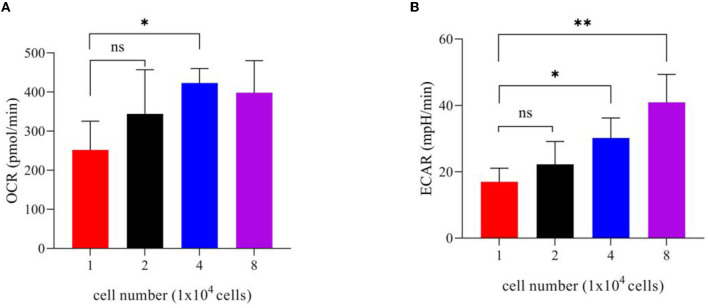
Bar chart of different cell numbers. **(A)** OCR values of different cell numbers. **(B)** ECAR values of different cell numbers. *P < 0.05 and **P < 0.01.

In the detection of mitochondrial pressure and glycolytic pressure, it was first necessary to determine the optimal cell seeding density (21). In this experiment, it is recommended that the basic oxygen consumption rate (OCR) value and the extracellular acidification rate (ECAR) be used to characterize the cell density. First, visual evaluation was used to approximate the optimal cell density: cells should be evenly distributed in each well with a fusion degree of 50–90%. On the one hand, when using a 24-well bioenergy analyzer to determine the cell OCR or ECAR values, the cell inoculation density should be 1 × 10^4^-8 × 10^4^ cells/well. On the other hand, the basic OCR and ECAR values can be used to determine the best sowing density and ensure the accuracy of the data. According to the product description of a Seahorse XFe24 analyzer, the cell OCR in the normal growth state should be in the range of 50–400 pmol/min and the normal basic ECAR range should be in the range of 20–120 mpH/min. Therefore, combining the two aspects, maintaining the cells at 8 × 10^4^/well will be more suitable (Figure 4) (21).

#### 3.4.2. Effect of TG on oxidative phosphorylation of HepG2 cells

**Figure 5:**
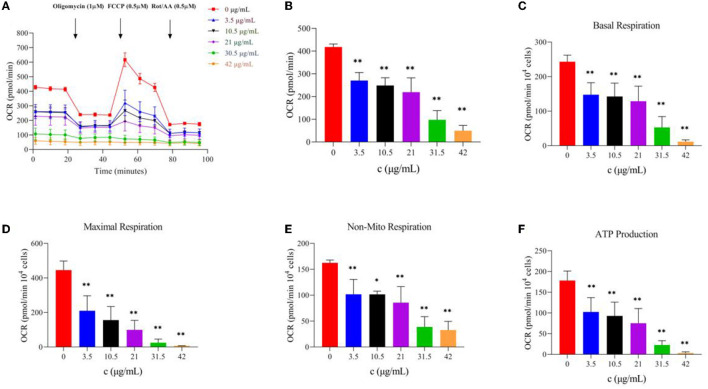
Effect of TG on oxidative phosphorylation of HepG2 cells. **(A)** OCR value curve at different time intervals. **(B)** OCR bar chart of TG with different concentrations. **(C)** Basic respiration at different TG concentrations. **(D)** Maximum respiration at different concentrations. **(E)** Non-mito respiration at different TG concentrations. **(F)** ATP production at different TG concentrations. *P < 0.05 and **P < 0.01.

The evaluation of mitochondrial function is essential for understanding the diseases related to cell energy metabolism and the development of corresponding drugs, and OCR is one of the most important evaluation indexes. To study the effect of TG on the oxidative phosphorylation process of HepG2 cells, we used a Seahorse XFe24 analyzer to detect the OCR levels of the cells treated with TG and analyzed the relevant indices. With an increase in TG concentration, the OCR level gradually decreased (Figures 5A, B), and especially, after the addition of FCCP, the difference in the OCR values between the dosing group and the control group became larger. At the same time, Figures 5C–F and Table 1 show that basal respiration decreased gradually; compared with 0 μg/ml, it decreased by 39.24%, 41.43%, 47.10%, 78.22%, and 95.26%, respectively, for each dosing group. The maximum respiration rate decreased by 52.95%, 65.06%, 77.76%, 94.48%, and 98.61%, respectively; Non-mitochondrial oxygen consumption decreased by 37.34%, 37.45%, 47.27%, 76.10%, and 79.79%, respectively. ATP production decreased by 42.49%, 47.91%, 57.83%, 87.28%, and 98.16%, respectively. The reasons for the decrease may be that the cells treated with TG were in the state of apoptosis and the level of ROS increased, which may increase the oxidative damage of the cells, reduce the activity of the mitochondrial respiratory chain complex, and affect the ability of mitochondrial ATP synthesis. The results showed that TG could significantly inhibit the overall level of aerobic respiration of cell mitochondria, and the cell survival rate after TG treatment was significantly reduced, which may be directly related to the disorder of mitochondrial energy metabolism. However, there is a need for more in-depth studies of the mechanisms.

**Table 1:** Parameters of mitochondrial oxidative phosphorylation.

**OCR (pmol/min)**	**Basal respiration**	**Maximal respiration**	**Non-mito respiration**	**ATP production**
0 μg/ml	243.04 ± 19.08	445.41 ± 52.32	162.36 ± 5.55	178.15 ± 22.99
3.5 μg/ml	147.66 ± 34.76^**^	209.55 ± 87.16^**^	101.73 ± 28.63^**^	102.46 ± 34.59^**^
10.5 μg/ml	142.36 ± 38.85^**^	155.64 ± 78.37^**^	101.55 ± 6.20^*^	92.8 ± 33.29^**^
21 μg/ml	128.58 ± 43.77^**^	99.07 ± 54.95^**^	85.61 ± 31.01^**^	75.13 ± 35.34^**^
31.5 μg/ml	52.94 ± 31.55^**^	24.59 ± 21.24^**^	38.8 ± 19.71^**^	22.66 ± 10.39^**^
42 μg/ml	11.52 ± 5.24^**^	6.18 ± 1.72^**^	32.81 ± 16.56^**^	3.27 ± 2.83^**^

^*^There is a significant difference (P < 0.05) between the treatment group and the 0 μg/ml group.

^**^P < 0.01.

#### 3.4.3. Effect of TG on the glycolytic ability of HepG2 cells

**Figure 6:**
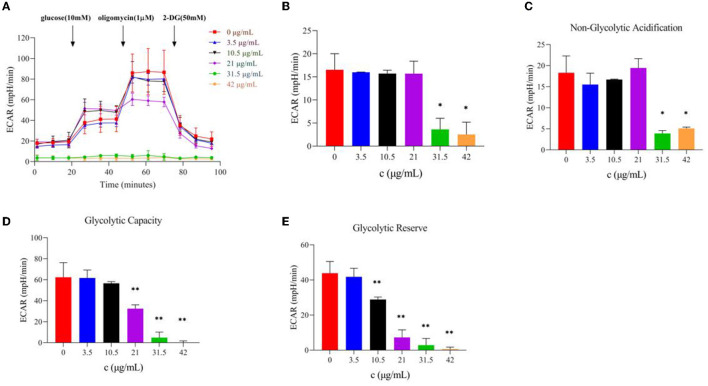
Effect of TG on the glycolytic function of HepG2 cells. **(A)** Curves of ECAR values at different times. **(B)** ECAR histogram of TG with different concentrations. **(C)** Non-glycolytic acidification with different TG concentrations. **(D)** The glycolytic capacity of TG at different TG concentrations. **(E)** Glycolytic reserve with different TG concentrations. *P < 0.05; **P < 0.01.

The glycolysis process is carried out in the cell matrix, and the produced lactic acid releases H^+^ to the outside of the cell. Moreover, a Seahorse XFe24 analyzer was used to analyze the glycolytic function by detecting the ECAR values after TG treatment and the relevant assay parameters to determine the specific method by which TG affects the glycolytic process of HepG2 cells. Over time, with the addition of glucose and oligomycin, the ECAR value increased gradually and showed a downward trend upon adding 2-DG (Figure 6A). With the increase in TG concentration, the overall ECAR level gradually decreased (Figure 6B), which was reflected by the gradual decline in glycolytic capacity and glycolytic reserve value (Figures 6D, E; Table 2). The non-glycosylation level was mostly lower than that of the control group but there was no concentration dependence (Figure 6C). Compared with the control group, the glycolytic capacity of the TG treatment group decreased by 1.12%, 9.29%, 34.46%, 92.15%, and 100.00%, respectively; the glycolytic reserve value decreased by 4.78%, 34.17%, 79.50%, 93.39%, and 98.63%, respectively. The results showed that TG could inhibit the proliferation and growth of HepG2 cells by reducing the glycolytic function of HepG2 and producing cytotoxicity. Of note, the damage degree of glycolytic function at low dose was lower than the decline degree of various parameters of cell oxidative phosphorylation, while glycolytic function and cell oxidative phosphorylation both were significantly reduced at high dose. Therefore, it was suggested that TG mainly damaged mitochondrial function at low dose, while at high dose both damaged mitochondrial function and glycolytic function at the same time, so as to accelerate the degree of apoptosis and necrosis of tumor cells and inhibit cell proliferation.

**Table 2:** Parameters of glycolytic stress.

**ECAR (mpH/min)**	**Non-glycolytic acidification**	**Glycolytic capacity**	**Glycolytic reserve**
0 μg/ml	18.3 ± 4.0	62.4 ± 13.9	43.9 ± 6.6
3.5 μg/ml	15.5 ± 2.7	61.7 ± 7.6	41.8 ± 4.9
10.5 μg/ml	16.7 ± 0.1	56.6 ± 1.6	28.9 ± 1.4^**^
21 μg/ml	19.4 ± 2.2	40.0 ± 3.7^**^	9.0 ± 1.7^**^
31.5 μg/ml	3.9 ± 0.7^*^	4.9 ± 5.2^**^	2.9 ± 3.8^**^
42 μg/ml	5.1 ± 0.3^*^	0.0 ± 1.7^**^	0.6 ± 1.1^**^

^*^There is a significant difference (P < 0.05) between the treatment group and the 0 μg/ml group.

^**^P < 0.01.

### 3.5. Expression of HepG2 cell proteins after TG treatment

**Figure 7:**
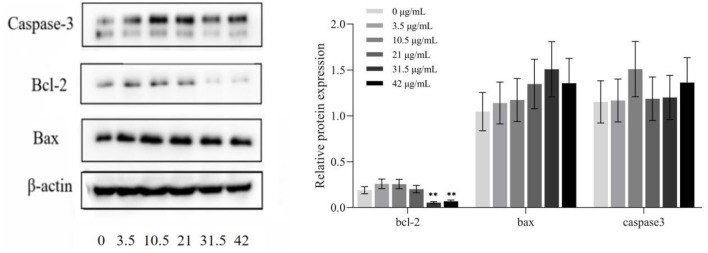
Protein expression in HepG2 cells treated with different concentrations of TG. *P < 0.05; **P < 0.01.

Figure 7 shows that, after treatment with different concentrations of TG for 24 h, the protein expression levels of HepG2 cells change concomitantly. As the concentration of TG increased, the expression levels of the proapoptotic factors Bax and Caspase-3 increased, and the relative protein expression was the highest when the concentrations were 31.5 μg/ml and 10.5 μg/ml, respectively. In addition, when the concentration was low (0, 3.5, 10.5, and 21 μg/ml TG), the relative expression of the anti-apoptotic factor Bcl-2 increased. When the TG concentration was 31.5 μg/ml and 42 μg/ml, it showed a significant downward trend. Therefore, we inferred that TG may promote the apoptosis of HepG2 cells.

## 4. Discussion and conclusion

Studies proved that *Cistanche deserticola* and its effective components have antioxidant, anti-fatigue, antiaging, antitumor, memory-improving properties and promote bone formation (11, 12). At present, the treatment for liver cancer still lacks effective means. It is often based on comprehensive treatment such as surgery, radiotherapy, and chemotherapy. Chemotherapy has large toxic and side effects and is easy to develop drug resistance. Traditional Chinese medicine has less effective antitumor toxicity and side effects. At the same time, it can enhance organic immunity and prevent cancer (24).

Cancer meets the biosynthesis and redox needs of tumor cells by changing the metabolism, tumor cell reprogramming, nutrient acquisition, and metabolic pathways, making it possible for tumor cells to proliferate without restriction (25). Therefore, restricting the proliferation of tumor cells through the above ways was the focus of our subsequent experiments. A certain concentration of TG was found to significantly inhibit the growth and proliferation of HepG2 cells in a dose-dependent manner with the increase in concentration.

Under normal circumstances, a slight increase in ROS level can promote the proliferation of normal cells. However, a significant ROS level increase in cancer cells can trigger cell death (26). High doses of ROS could cause cell growth inhibition, injury, apoptosis, and even death, which was consistent with the inhibitory effect of TG on the proliferation of HepG2 cells. There is also evidence that ROS production usually inhibits the proliferation of cancer cells (27). The survival or death of cells largely depends on the functional state of mitochondria (28). ROS is an inevitable product in the process of cell metabolism as it acts on mitochondria and is one of the main ways to cause mitochondrial damage. The main function of mitochondria in cells is to provide energy for cell metabolism and biosynthesis by producing ATP, which is very important for the survival of normal cells and tumor cells (29). If the ROS level increases in the body and is not decomposed by cells, ROS will affect the activity of the mitochondrial respiratory chain complex (30) and, thus, affect the oxidative phosphorylation of mitochondria and reduce ATP synthesis in cells (31), resulting in the imbalance of the intracellular redox system and inhibition of the proliferation of tumor cells (32). Indeed, chemopreventive agents could enhance the level of ROS to reach the toxicity threshold and promote cancer cell apoptosis with the least toxicity to normal cells (33). The results also confirmed that the increase in the relative content of ROS could significantly reduce the survival rate of HepG2 cells.

To adapt to changing external environments, an organism must adjust the biological activities of cells at any time. Correspondingly, the regulation of cell biological activities by the organism is based on its regulation of cellular metabolic networks. Therefore, the energy state of cells is always regulated and maintained in a certain range of dynamic balance under normal physiological conditions, which is the steady state of cell energy metabolism (34). In addition, the mitochondria are considered one of the main targets of drug-induced hepatocyte injury (35). At the same time, researchers believe that the energy needs of cells can be met through glycolysis and oxidative phosphorylation (OXPHOS). When OXPHOS is no longer available, for example, due to hypoxic effects or mitochondrial damage, these cell lines can switch to glycolysis to produce sufficient ATP for continued survival (36).

Otto Warburg (37, 38) observed that, in the presence of oxygen, tumor cells exhibited unusual characteristics of absorbing glucose and fermenting it into lactic acid. This characteristic, aerobic glycolysis indicates that, with sufficient oxygen, tumor cells preferred to use glycolysis for glucose metabolism instead of mitochondrial oxidative phosphorylation to produce more ATP. This will help tumor cells to quickly produce enough energy for their rapid growth, release more lactic acid, maintain their acidic microenvironment, escape immune surveillance, and easily metastasize (39, 40). Therefore, Warburg proposed that mitochondrial respiratory defects are a potential basis for aerobic glycolysis and cancer (37, 38). In this study, all concentrations of TG significantly inhibited the overall level of aerobic respiration of mitochondria and limited the glycolytic function, which may be directly related to the disorder of mitochondrial energy metabolism. However, there is a need for more in-depth studies of the mechanisms.

Bax is the main regulator of Bcl-2 activity and its expression levels are directly related to apoptosis regulation, with increased Bax expression indicating the promotion of apoptosis by drugs and increased Bcl-2 indicating the inhibition of apoptosis (41). In addition, caspase protease highly influences apoptosis and its activation represents the progression of cells into the irreversible apoptosis phase (42). Thus, in HepG2 cells treated with TG, the extent of caspase-3 activation was clearly increased, indicating that TG could cause irreversible apoptosis and prevent cell proliferation.

In conclusion, this study has significant reference to in-depth research on the anti-tumor mechanism of *Cistanche deserticola*. It has the potential to be a raw material of drugs for the clinical treatment of liver cancer and to effectively prevent tumors with consumption in daily diet.

## Data availability statement

The datasets presented in this study can be found in online repositories. The names of the repository/repositories and accession number(s) can be found in the article/Supplementary material.

## Author contributions

DF: investigation, writing original draft, and plot analyses. S-qZ and Y-xZ: formal analysis and visualization. Y-jJ: investigation. Q-dS, WS, and Q-qC: resources and material support. W-jY: writing review and editing, project supervision, and funding acquisition. JW: writing review and editing and validation. All authors approved the final version of the manuscript.
